# Comparison of transoral endoscopic thyroidectomy vestibular approach and open conventional thyroidectomy regardıng inflammatory responses, pain, and patient satisfaction: a prospective study

**DOI:** 10.3389/fsurg.2023.1281093

**Published:** 2023-11-16

**Authors:** Mehmet Taner Ünlü, Nurcihan Aygun, Erdinc Serin, Mehmet Uludag

**Affiliations:** ^1^ Department of General Surgery, Sisli Hamidiye Etfal Training and Research Hospital, Istanbul, Turkey; ^2^ Department of Biochemistry, Sisli Hamidiye Etfal Training and Research Hospital, Istanbul, Turkey

**Keywords:** open thyroidectomy, TOETVA, postoperative pain, cosmesis, complications, comparison

## Abstract

**Introduction:**

The application of transoral endoscopic thyroidectomy vestibular approach (TOETVA) is becoming widespread throughout the world. We primarily aimed to evaluate the severity of surgical trauma in TOETVA and conventional open thyroidectomy (COT) regarding the inflammatory response including the comparison of surgical stress markers [interleukin-6 (IL-6), C-reactive protein (CRP), white blood cell (WBC)].

**Material and method:**

This non-randomized prospective study enrolled two groups with 20 patients each: COT group and TOETVA group. Patients aged 18–65 years with benign thyroid disease; with fine needle aspiration biopsy results of Bethesda III, IV or Bethesda V, VI (<1 cm nodule); thyroid volume <50 cm^3^; nodule diameter <4 cm; female gender without a previous neck, chin, and/or oral surgery; without vocal cord paralysis preoperatively; and patients in euthyroid state were enrolled to the study. Preoperative, postoperative second hour, first day, and second day CRP, WBC, and IL-6 levels were evaluated. Pain intensity was evaluated with the visual analog scale (VAS) score on the 2nd and 12th hour, 1st and 2nd days postoperatively.

**Results:**

All the patients were female and mean age was significantly higher in the COT group. The operative time was significantly longer in the TOETVA group. No significant difference was found between the two groups regarding IL-6 levels. In the TOETVA group, postoperative second hour WBC value (*p* = 0.044) and first (*p* = 0.002) and second day (*p* = 0.005) CRP values were significantly higher. In the TOETVA group, the lower lip and lower chin VAS scores were significantly higher at 2nd and 12th hour, on the first and second days. The anterior neck VAS score was significantly higher in the TOETVA group at the second hour (*p* = 0.025). General and cosmetic satisfactions were similar at the 15th and 30th days in both groups.

**Conclusion:**

The longer operative time and higher postoperative CRP level and VAS score in the chin and lower lip in the TOETVA group suggested that the method is not a minimally invasive technique compared to COT. However, the presence of similar total complication rates and early postoperative general and esthetic satisfaction that improves over time in both groups suggests that the clinical effect of increased magnitude of systemic inflammatory response in TOETVA might be temporary and acceptable.

## Introduction

Thyroidectomy is the most common endocrine surgical procedure and the standard approach is open thyroidectomy via cervical incision (1). Conventional open thyroidectomy (COT) leaving an incision scar of 5–10 cm in the neck has an esthetically negative effect on the patient population consisting of mostly young females, which may lead to psychological trauma and reduce the quality of life (2). Minimally invasive surgery can be defined as the novel traditional surgical procedure, which can be performed in new ways that would leave the surgical field with minimal trauma (3).

Minimally invasive surgery, which started with the first laparoscopic cholecystectomy approximately 30 years ago, has replaced many conventional surgeries today and is among the standard treatment approaches. Techniques defined as minimally invasive, such as total endoscopic thyroidectomy and minimally invasive video-assisted thyroidectomy, were introduced in thyroid surgery in the 1990s. However, these techniques also leave a visible incision scar on the neck, albeit small (2).

In the past two decades, remote access endoscopic or robotic thyroidectomy methods such as transaxillary approach and axillo-breast approach have been popular (4). These techniques do not cause a scar in the neck, but the incisions are moved to the areas that can be covered with clothing to eliminate the need for a smaller incision. These methods are not minimally invasive as a wide dissection of the chest and neck is required to reach the thyroid (2, 5, 6). After transoral thyroidectomy, a natural orifice transluminal endoscopic surgical technique was developed by Witzel et al. in 2008, and different transoral thyroidectomy techniques were tried (7). In 2016, after Anuwong reported that the transoral endoscopic thyroidectomy vestibular approach (TOETVA) is a safe and feasible technique, it became popular. The application of the TOETVA technique is becoming widespread throughout the world (8). TOETVA is a minimally invasive procedure compared to other remote access thyroidectomies because it requires less flap dissection to reach the thyroid gland and is a true scarless technique (9).

However, it is not clear whether TOETVA is a real minimally invasive technique compared to COT, requiring three incisions in the vestibulum, and more subplatysmal flap dissection to reach the thyroid gland than COT requires. All minor and major surgeries cause systemic inflammatory response (SIR) due to operative injuries in the body. In clinical practice, cortisol, interleukin-6 (IL-6) (a proinflammatory cytokine), white blood cell (WBC), and C-reactive protein (CRP) are the most commonly used parameters to evaluate the magnitude of the SIR. SIR severity after elective surgery is related to the extent of operative injury and invasiveness of the operation (10). In addition to SIR, pain score, operative time, postoperative drainage volume, and length of the postoperative hospital stay can be used to evaluate the level of surgical trauma (11, 12).

Despite the significant differences in the surgical site, localization and length of incision, and tissue retracting between COT and TOETVA, surgical stress markers (IL-6, CRP, and WBC) can be used to compare the magnitude of SIR as a quantifiable method to evaluate the surgical stress.

During the planning period of the present study, there has been no study in the literature evaluating the surgical stress markers related to the severity of surgical trauma in TOETVA. The research question of this study is whether the severity of surgical stress in TOETVA is less than COT or not.

In the present study, as a primary end-point, we aimed to evaluate the severity of surgical trauma in TOETVA and COT regarding the inflammatory response including the comparison of surgical stress markers (IL-6, CRP, and WBC). We also evaluated the postoperative pain, operative time, and hospital stay as secondary end-points.

## Material and methods

### Study design and patients

This non-randomized prospective clinical study was performed in our clinic between September 2019 and January 2020 with the approval of the Şişli Hamidiye Etfal Training and Research Hospital Ethics Committee (Ethics committee date: 19/03/2019, number: 2313). In addition, support was received from the Health Science University, scientific research projects coordinator with project no 2019/067.

The sample size was calculated with G*Power 3.1.9.3. Since there has been no study in the literature comparing TOETVA and COT regarding the inflammatory response related to the severity of the surgical stress, the sample size was found as a total of 34 patients (17 for each group) considering the alpha margin of error as 0.05, power (1-Beta): 0.80, effect size: 1, N1/N2 = 1/1 for calculating the sample size. The sample size was decided as 40 patients with 20 per group.

### Inclusion criteria

Patients aged 18–65 years with benign thyroid disease; with fine needle aspiration biopsy (FNAB) results of Bethesda III, IV or Bethesda V, VI (below 1 cm nodule); thyroid volume less than 50 cm^3^; nodule diameter less than 4 cm; female gender without a previous neck, chin, and/or oral surgery; without vocal cord paralysis (VCP) preoperatively; clinically euthyroid and laboratory results in the euthyroid state; and who agreed to participate in the study were enrolled in the study. Female gender was preferred because male patients may have more difficult passage from the chin to the neck due to the more robust tissue. Male gender's thyroid cartilage is larger and protruding, preventing the endoscopic vision and manipulation of the instruments, which might prolong the operation time and affect the severity of traumatic injury.

### Exclusion criteria

Patients with an FNAB result of Bethesda V, VI over 1 cm nodule in size, planning of central and/or lateral neck dissection due to thyroid cancer, thyroid volume greater than 50 cm^3^, secondary intervention cases, patients with accompanying hyperparathyroidism, patients with preoperative VCP, and patients with ASA score of III and IV were not included. Patients with conversion from TOETVA to the conventional method intraoperatively were also excluded from the study.

### Creating groups

The study enrolled two groups including 20 patients in each: COT group (Group 1) and TOETVA group (Group 2). All patients who agreed to participate in the study were informed about both surgical techniques and complications, and written consent was obtained. Patients took part in Group 1 or Group 2 according to their own preferences.

**Figure 1:**
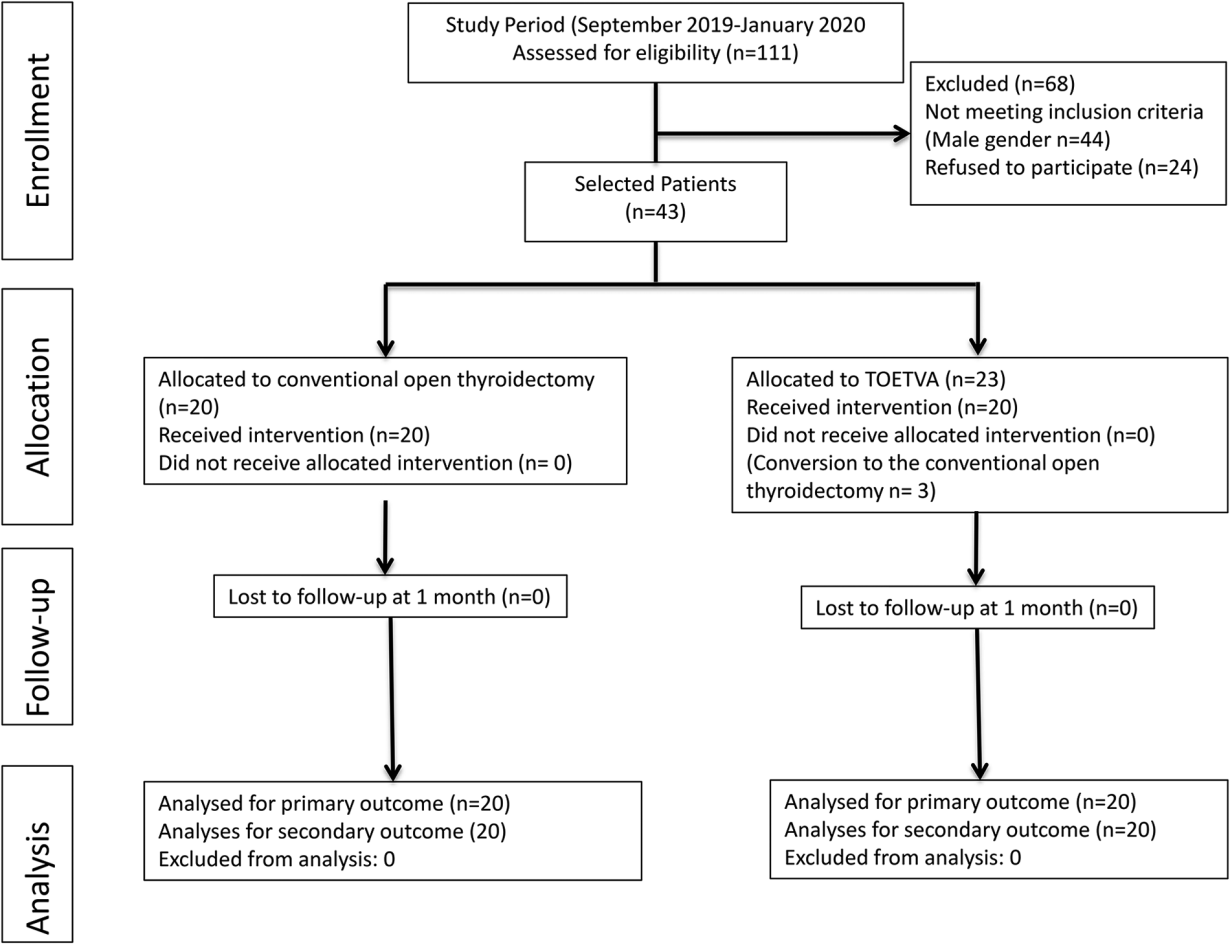
Flowchart of study.

As presented in the flowchart (Figure 1), during this period, 111 patients underwent thyroidectomy. Patients who did not comply with the study criteria were excluded from the study. About 67 female patients were selected out of those who were eligible for the study. Of these patients, 24 did not agree to participate in the study. Three patients whose surgical procedures were converted to open surgery were excluded from the study, and a total of 40 patients were included in the study.

### Surgical technique

All surgeries were performed by an experienced endocrine surgeon (MU).

### Intraoperative neuromonitorization technique

The NIM 3.0 Nerve Monitoring System (Medtronic Xomed, Jacksonville, FL, USA) including endotracheal surface electrodes was used to check the nerve intraoperatively. General anesthesia was administered to each patient with a low-dose short-acting neuromuscular blockade (rocuronium 0.3 mg/kg) and intubated with a Medtronic Xomed Nerve Integrity Monitor Standard Reinforced Electromyography Endotracheal Tube (size 6.0, 7.0, or 8.0). Standard intraoperative neural monitoring (IONM) technique was performed as a four-step procedure (V1, R1, R2, and V2). The IONM setup, applications, and data interpretation were applied in compliance with International Neural Monitoring Guidelines (13, 14). Vocal cord examination was performed preoperatively and between postoperative 24th–48th hours for all patients.

### Conventional open thyroidectomy

After neck extension with a thyroid pillow and patient positioning, thyroidectomy and/or central neck dissection was performed using a 4 to 6 cm collar transverse incision. Subplatysmal flap dissection was limited to the sternal notch inferiorly and the thyroid cartilage superiorly, and through the midline of the strap muscles, the thyroid gland was reached. After receiving a positive signal from the vagus nerve, upper pole dissection was performed under the guidance of IONM. Identification and monitoring of recurrent laryngeal nerves (RLNs) and external branch of the superior laryngeal nerves (EBSLNs) were carried out systematically, as described previously (15, 16). The upper pole vessels were divided on the thyroid capsule with Ultracision® or bipolar cautery. With lateral approach, RLN was identified in the region where it crosses the inferior thyroid artery (ITA). Then, RLN was exposed till its laryngeal entry under the cricopharyngeal muscle (CPM). Preserving parathyroids, ITA branches were divided from the capsule. R2 and V2 signals were obtained after bleeding control. Surgicel® was placed in the surgical field and strap muscles were reapproximated with 3/0 polyglactin. The subcutaneous tissue was sutured separately with polyglactin. The skin was reapproximated subcutaneously with polyglactin.

### Transoral endoscopic thyroidectomy vestibular approach

All patients received preoperative chlorhexidine mouthwash and preoperative intravenous amoxicillin/clavulanic acid was administered for prophylaxis. Orotracheal intubation was performed. A slight extension posture was given to the neck with a pillow placed under the shoulder, and the patients were placed in 15° in the Trendelenburg position. Skin and oral cavity were wiped with povidone iodine. A central 1.5–2 cm transverse incision was performed in the middle of the distance between the first vermillion inner edge and the inferior labial frenulum in the oral vestibule. Through the peripheral fibers of the submucosa and orbicularis oris muscle, the jaw tip was reached with monopolar electrocautery between the two mentalis muscles deep in the subdermal layer. Through this incision, 50 cc of 1/500,000 adrenaline-saline solution was applied to the anterior neck with a Veress needle. A surgical field was created through this incision toward the thyroid cartilage via dissection with Kelly forceps. The surgical field was formed by blunt dissection using a blunt-type tunnel probe in the subplatysmal area, limited to anterior edges of sternocleidomastoid muscles (SCMs) laterally and to the sternal notch inferiorly. A 10-mm blunt-tipped port was entered through the central incision to insert the 30° camera and operation was performed under 6 mmHg CO_2_ pressure with a 15 L/min CO_2_ flow rate. In addition, with three or four silk sutures placed on the front neck, the skin is mechanically hung and an optimal working area is provided. Two vertical 5 mm incisions were made at the level of canine tooth and on the edges of vermillion at both sides, and two working ports were placed parallel to the 10 mm port. The subplatysmal workspace was opened completely with hook cautery and harmonic scalpel. Strap muscles were opened in the midline and dissected over the thyroid gland. The strap muscles were retracted by a transcutaneous 2/0 silk sutures. Thyroid isthmus is divided. The upper pole was dissected, the EBSLN was checked with the monitoring probe, and the thyroid vessels were divided from the thyroid capsule with Ultracision. The superior parathyroid gland was identified and dissected carefully. The RLN was identified at the laryngeal entry and mapped proximally. The Berry fibers were divided on the thyroid capsule to protect the RLN. The inferior parathyroid gland was identified and protected. The thyroid gland was separated from the trachea and placed in the endobag inserted through the 10 mm port and then it was extracted. The same surgical procedures were applied to the opposite lobe in total thyroidectomy. Following bleeding control, RLN (R2) and vagus (V2) signals were obtained using the IONM probe and recorded. A Surgicel was placed into the surgical field. The SCMs were approximated with 3/0 polyglactin sutures. No drain was used in any patient. Intraoral incisions were sutured with 4/0 polyglactin. A 24-h pressure dressing was applied over the chin.

### Inflammatory response

Preoperative, postoperative second hour, first day, and second day CRP (normal range: <5 mg/L), WBC (normal range: 4.5–10.5 × 10^9^/L), and IL-6 (normal range: <7 ng/L) levels were measured to evaluate the inflammatory response in all patients. To measure IL-6, blood samples were collected from patients, centrifuged, and stored at −80°C. After all the serums were collected, they were evaluated using the Human IL-6 ELISA kit. To evaluate postoperative hypoparathyroidism, postoperative first day calcium, phosphorus, and parathormone values were checked. Hypocalcemic patients were controlled weekly and evaluated both clinically and with laboratory values.

### Postoperative pain and analgesia

Routinely, in terms of pain palliation, all patients received 4 × 1,500 mg paracetamol intravenously and 3 × 1,100 mg tramadol HCL on the first day; and 3 × 1,500 mg paracetamol intravenously on the second day in the postoperative period.

Pain intensity of the patients was evaluated with the visual analog scale (VAS) score (0–10) on the second and 12th hour, first, and second days postoperatively. Lower lip, lower chin, and anterior neck area pain were evaluated separately using the VAS scale. VAS scores were determined by patients marking the intensity of pain on the ruler, demonstrating 0 for the absence of pain and 10 for the most severe pain sensation (17). No patient was given extra analgesic.

Patient satisfaction was evaluated on postoperative 15th and 30th days in both groups. Patients were asked to evaluate and score the operation in general and in terms of cosmesis (1: bad and 4: very good).

Vocal cord examination with fiber optic laryngoscopy was performed to all patients in the preoperative period and within the first two days postoperatively by an independent otorhinolaryngologist. Control examinations were planned for patients with VCP at the 15th day, 1st, 2nd, 4th, and 6th months postoperatively.

Intraoperative and postoperative complications of patients were recorded.

### Statistical analysis

Data analysis was performed with IBM SPSS Statistics Version 25. Non-parametric tests were preferred in all comparisons, since the number of cases in groups was less than 30. Mann–Whitney U test, Chi-squared test, Wilcoxon signed-rank test, and McNemar tests were used in comparisons between groups. *P* < 0.05 was considered statistically significant. Subgroup analyses of each groups (Group 1 and Group 2) were calculated with Kruskal–Wallis one-way ANOVA test, pair-wise comparisons were calculated with independent-samples Kruskal–Wallis test, significance values have been adjusted by the Bonferroni correction for multiple tests.

## Results

All the patients in the present study were female and the mean age was significantly higher in the COT than in the TOETVA group (50.3 ± 6.2 years vs. 42.9 ± 9.7 years, respectively, *p* = 0.008) (Table 1). There was no significant difference between the two groups in terms of height, weight, body mass index (BMI), thyroid volume, largest nodule diameter, type of surgery, number of operated lobes, and duration of hospital stay (Table 1).

**Table 1:** General characteristics of two groups, duration of surgery and hospital stay.

		TOETVA	COT	*p*
Age	Mean ± SD Min–max	42.9 ± 9.7 18–57	50.3 ± 6.2 34–59	0.008
BMI	Mean ± SD Min–max	26.6 ± 4.6 20.2–36.6	27.9 ± 4.2 19.7–37.2	0.267
Preoperative diagnosis (*n*)				
	MNG	9	13	0.349
	Basedow Graves	5	2	
	Bethesda III and IV	6	5	
Postoperative diagnosis (*n*)				
	Adenomatous Hyperplasia	13	13	0.856
	Lymphocytic Thyroiditis	3	2	
	Malignity	4	5	
Thyroid volume (cm^3^)	Mean ± SD Min—max	17.8 ± 10.6 2.9–46.9	24.6 ± 12.0 5.2–49.5	0.479
Largest nodule diameter (mm)	Mean ± SD Min–max	18.8 ± 12.2 6–46	27.4 ± 16.2 10–50	0.116
Surgery type				0.736
	Lobectomy	7	6	
	Total thyroıdectomy	13	14	
Operated lobe				
	Rıght	19	19	0.889
	Left	14	15	
Operation time (min)				
Total thyroidectomy	Mean ± SD Min–max	195 ± 51.4 105–300	99.6 ± 29.8 60–140	<0.001
Lobectomy	Mean ± SD Min–max	112.9 ± 34 80–170	49.2 ± 17.7 30–70	0.003
Duration of hospital stay (days)		2.5 ± 1.8 2–10	2 ± 0 2–2	0.076

Min, minimum; Max, maximum; SD, standard deviation; *n*, number; MNG, multinodular goiter.

The operation time in the TOETVA group was significantly longer in patients undergoing both lobectomy (113 ± 34 vs. 49 min, *p* = 0.003) and total thyroidectomy (195 ± 51 vs. 100 ± 30, *p* < 0.001) (Table 1).

### Systemic inflammatory response (traumatic inflammatory response)

**Figure 2:**
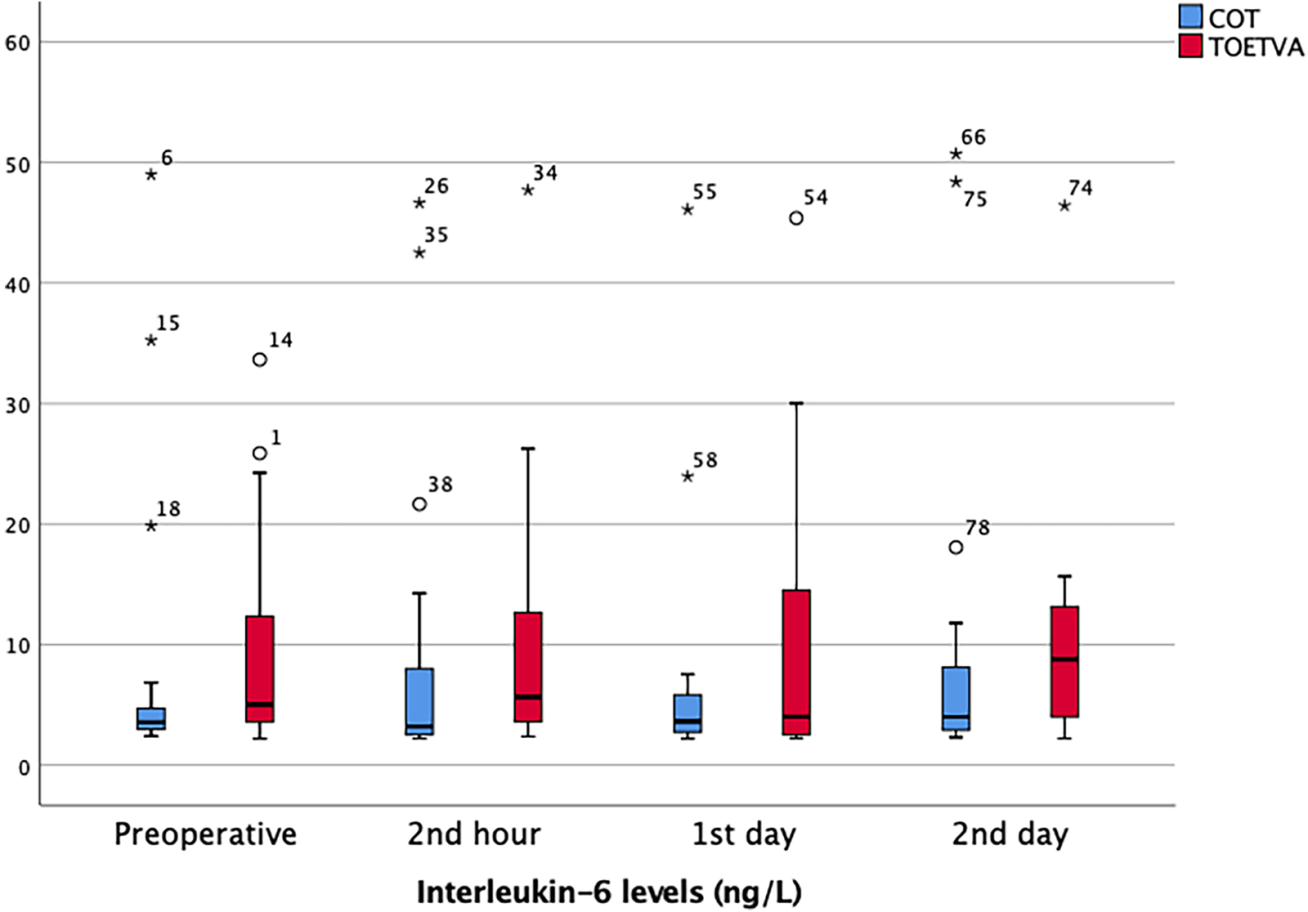
IL-6 results in graphical illustrations for both groups. *, extreme outlier; o, mild outlier; minimum value that is not an outlier, represented by the bottom of the vertical line (whisker) that extends from the bottom of the box; maximum value that is not an outlier, represented by the top of the vertical line (whisker) that extends from the top of the box; first quartile (Q1), the value below which 25% of the values in the data set are found; median, the value that separates the higher half of the data set from the lower half; third quartile (Q3), the value below which 75% of the values in the data set are found; interquartile range (IQR), the box in the boxplot. This is the middle 50% of the data set (between Q1 and Q3).

IL-6 (<7 ng/L): Preoperative, postoperative second hour, first, and second day IL-6 levels were similar, and no significant difference was found between the two groups (Table 2) (Figure 2).

**Table 2:** IL-6, CRP, and WBC levels.

	Time	TOETVA	COT	*p*
IL-6 (ng/L) Mean ± SD Min–max	Preoperative	8.3 ± 12.4 2.4–49	9.5 ± 9.2 2.18–33.64	0.168
	Second hour	9.2 ± 13.1 2.2–46.6	9.9 ± 10.8 2.4–47.7	0.091
	First day	7 ± 10.3 2.2–46.1	9.4 ± 11.2 2.2–45.4	0.607
	Second day	9.8 ± 14.1 2.3–50.7	10. 2 ± 9.6 2.2–46.4	0.130
WBC (10^9^/L) Mean ± SD Min–max	Preoperative	6.8 ± 2^a^ 3.8–11.6	7.2 ± 1.6^e^ 4.1–10.6	0.425
	Second hour	13.1 ± 4.8^b^ 4.4–21.6	10.2 ± 4^f^ 5.2–19.3	0.044
	First day	10.7 ± 2.5^c^ 6.2–14.8	10 ± 3^g^ 5.4–15.3	0.449
	Second day	8.8 ± 1.9^d^ 4.8–12.9	9.2 ± 2.7^h^ 4.2–14.6	0.607
CRP (mg/L) Mean ± SD Mın–max	Preoperative	2.9 ± 2.5^a1^ 0.4–9.4	3.2 ± 4^e1^ 0.5–18	0.935
	Second hour	4.1 ± 5^b1^ 0.6–21.6	4.1 ± 4.9^f1^ 0.6–18.1	0.957
	First day	54.3 ± 36^c1^ 6.1–157.6	32.6 ± 37.9^g1^ 5.7–146.3	0.002
	Second day	79.4 ± 54.6^d1^ 21.1–199.4	44.1 ± 39.9^h1^ 12.7–181.3	0.005

Min, minimum; Max, maximum; SD, standard deviation.

Group 1 WBC (*p* < 0.001, Kruskal–Wallis test) adjusted by Bonferroni correction for multiple tests: a vs. b: *p* < 0.001, a vs. c: *p* < 0.001, b vs. d: *p* = 0.022; group 2 WBC (*p* = 0.011, Kruskal–Wallis test) adjusted by Bonferroni correction for multiple tests: e vs. f: *p* = 0.044, e vs. g: *p* = 0.017; group 1 CRP (*p* < 0.001, Kruskal–Wallis test) adjusted by Bonferroni correction for multiple tests: a1 vs. c1: *p* < 0.001, a1 vs. d1: *p* < 0.001, b1 vs. c1: *p* < 0.001, b1 vs. d1: *p* < 0.001; group 2 CRP (*p* < 0.001, Kruskal–Wallis test) adjusted by Bonferroni correction for multiple tests: e1 vs. g1: *p* < 0.001, e1 vs. h1: *p* < 0.001, f1 vs. g1: *p* < 0.001, f1 vs. h1: *p* < 0.001.

**Figure 3:**
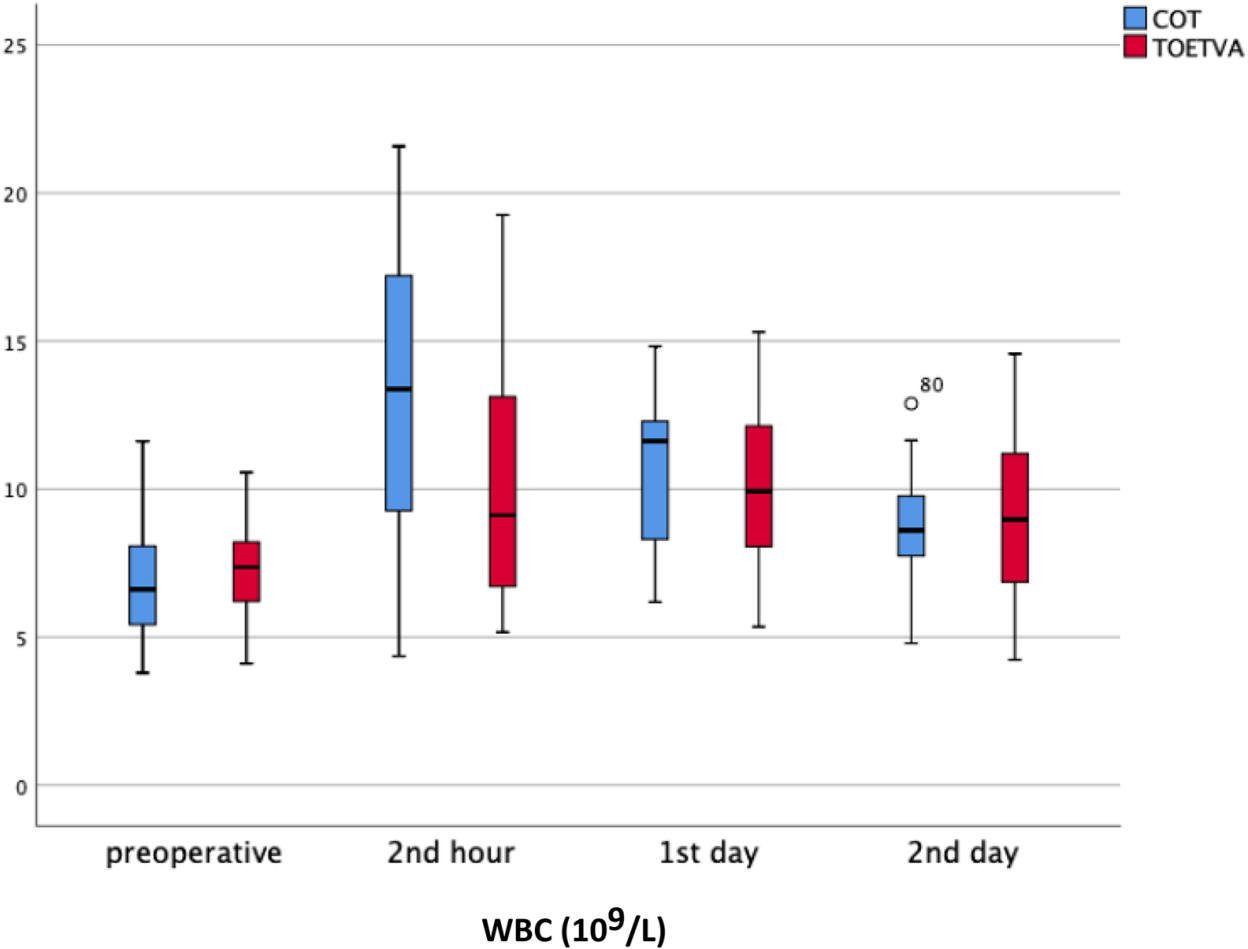
WBC results in graphical illustrations for both groups. o, mild outlier; minimum value that is not an outlier, represented by the bottom of the vertical line (whisker) that extends from the bottom of the box; maximum value that is not an outlier, represented by the top of the vertical line (whisker) that extends from the top of the box; first quartile (Q1), the value below which 25% of the values in the data set are found; median, the value that separates the higher half of the data set from the lower half; third quartile (Q3), the value below which 75% of the values in the data set are found; IQR, the box in the boxplot. This is the middle 50% of the data set (between Q1 and Q3).

WBC (4.5–10.5 × 10^9^/L): Preoperative WBC values were similar and postoperative second hour WBC value was significantly higher in the TOETVA group (13.1 ± 4.8 vs. 10.2 ± 4, *p* = 0.044). There was no difference between the two groups regarding WBC values on postoperative day 1 and day 2 (Table 2) (Figure 3). In both groups (COT and TOETVA), WBC values at second hour (*p* < 0.001, *p* = 0.044, respectively) and day 1 (*p* < 0.001, *p* = 0.017, respectively) were significantly higher than preoperative WBC values. Day 2 values were similar to preoperative values (*p* = 0.147, *p* = 0.122, respectively).

**Figure 4:**
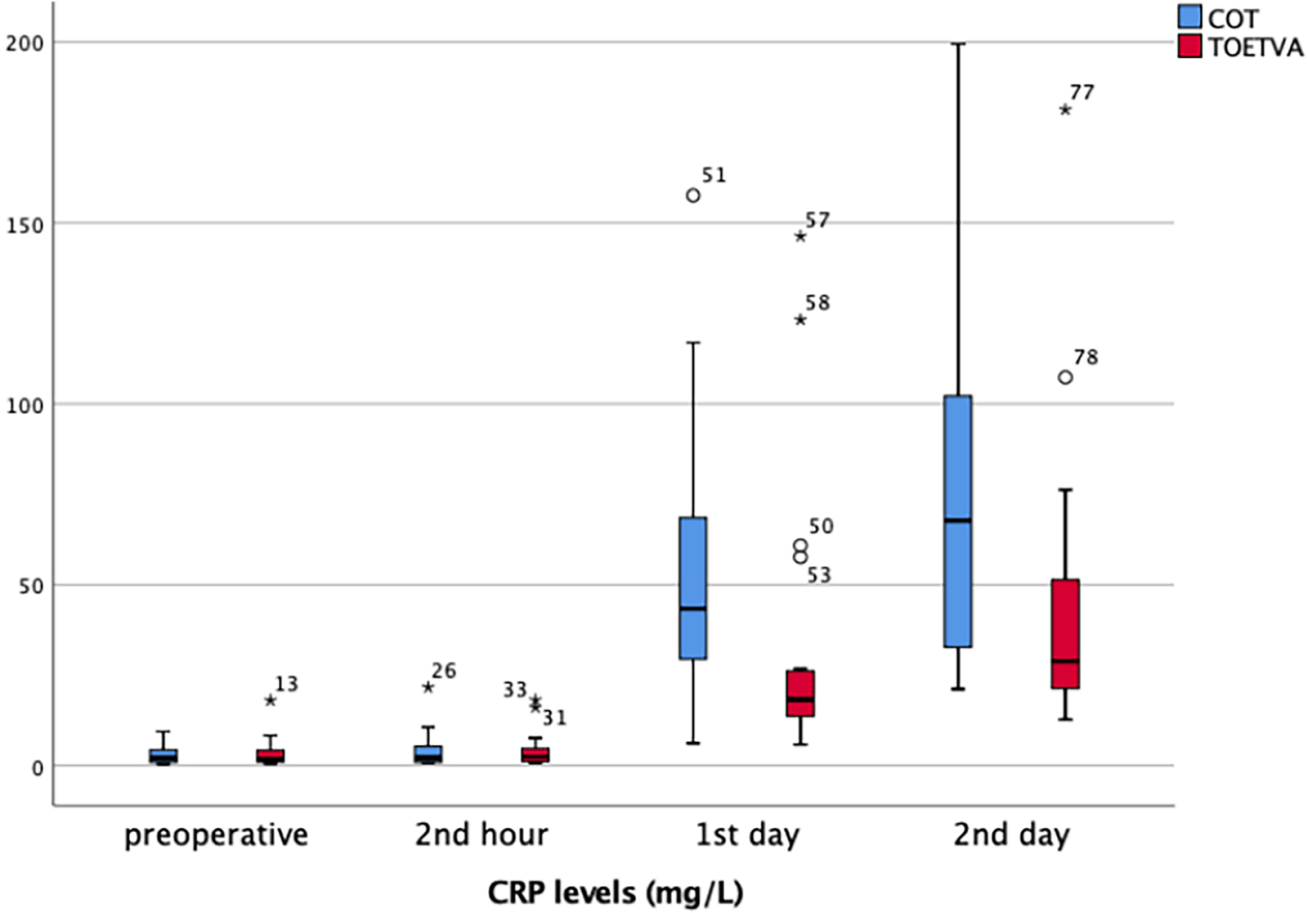
CRP results in graphical illustrations for both groups. *, extreme outlier; o, mild outlier; minimum value that is not an outlier, represented by the bottom of the vertical line (whisker) that extends from the bottom of the box; maximum value that is not an outlier, represented by the top of the vertical line (whisker) that extends from the top of the box; first quartile (Q1), the value below which 25% of the values in the data set are found; median, the value that separates the higher half of the data set from the lower half; third quartile (Q3), the value below which 75% of the values in the data set are found; IQR, the box in the boxplot. This is the middle 50% of the data set (between Q1 and Q3).

CRP (<5 mg/L): Preoperative and postoperative second hour CRP values were similar between the two groups. CRP values were significantly higher on the postoperative day 1 (54.3 ± 36 vs. 32.6 ± 37.9, *p* = 0.002) and day 2 (79.4 ± 54.6 vs. 44.1 ± 39.9, *p* = 0.005) in the TOETVA compared to the COT group (Table 2) (Figure 4). An increase in CRP over time was noted in both groups, and CRP values were higher in the TOETVA group. In COT, CRP values were found to be significantly higher on day 1 (*p* < 0.001, *p* < 0.001, respectively) and day 2 (*p* < 0.001, *p* < 0.001, respectively) compared to preoperative and postoperative second hour CRP values. Similarly, in the TOETVA group, day 1 (*p* < 0.001, *p* < 0.001, respectively) and day 2 CRP values (*p* < 0.001, *p* < 0.001, respectively) were significantly higher than those of preoperative and postoperative second hour.

### Evaluation of postoperative pain

#### Lower lip

**Figure 5:**
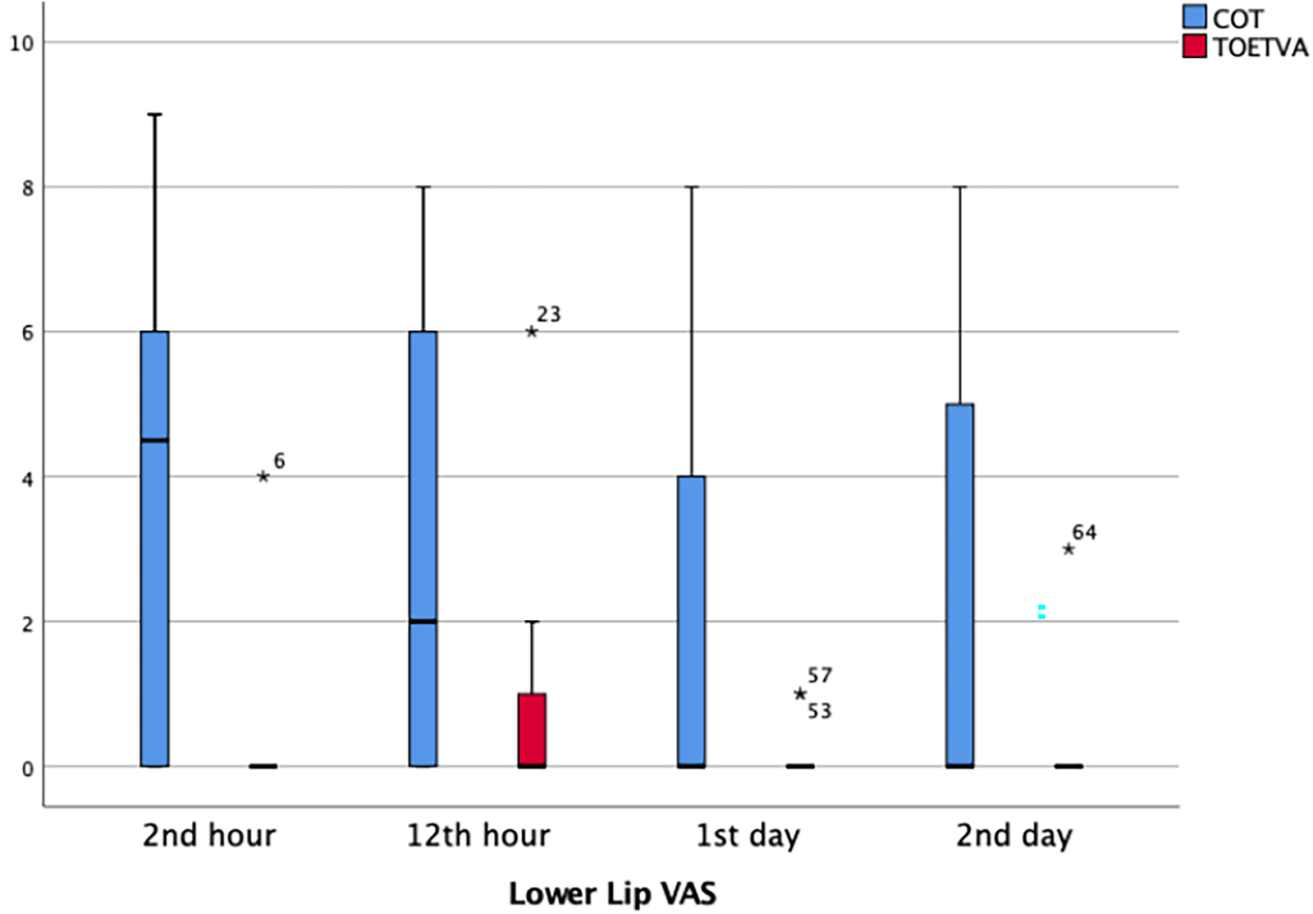
Lower Lip VAS score results in graphical illustrations for both groups. *, extreme outlier; minimum value that is not an outlier, represented by the bottom of the vertical line (whisker) that extends from the bottom of the box; maximum value that is not an outlier, represented by the top of the vertical line (whisker) that extends from the top of the box; first quartile (Q1), the value below which 25% of the values in the data set are found; median, the value that separates the higher half of the data set from the lower half; third quartile (Q3), the value below which 75% of the values in the data set are found; interquartile range (IQR), the box in the boxplot. This is the middle 50% of the data set (between Q1 and Q3).

Lower lip VAS scores were significantly higher in the TOETVA than in the COT group at postoperative second hour (*p* < 0.001), 12th hour (*p* = 0.016), and second day (*p* = 0.030). Although the lower lip VAS score was higher in the TOETVA on the postoperative first day, the difference was not statistically significant (1.7 ± 2.8 vs. 0.1 ± 0.3, *p* = 0.075) (Table 3) (Figure 5).

**Table 3:** Postoperative lower lip, lower chin and anterior neck pain VAS scores.

	Time	TOETVA	COT	*p*
Lower lip VAS score Mean ± SD Min–max	Postop second hour	3.9 ± 3.4 0–9	0.2 ± 0.9 0–4	<0.001
	Postop 12th hour	2.9 ± 3 0–8	0.7 ± 1.5 0–6	0.016
	Postop first day	1.7 ± 2.8 0–8	0.1 ± 0.3 0–1	0.075
	Postop second day	2 ± 3.2 0–8	0.2 ± 0.7 0–3	0.030
Lower chin VAS score Mean ± SD Min–max	Postop second hour	5.1 ± 3.4 0–9	0.7 ± 1.8 0–6	<0.001
	Postop 12th hour	4.6 ± 2.4 0–8	0.8 ± 1.6 0–6	<0.001
	Postop first day	4.2 ± 2.9 0–8	0 ± 0 0–6	<0.001
	Postop second day	3.5 ± 3.1 0–8	0.6 ± 2 0–8	0.001
Anterior neck VAS Score Mean ± SD Min–max	Postop second hour	7.7 ± 2.1 2–10	6 ± 2.7 0–10	0.025
	Postop 12th hour	6.8 ± 2.7 0–10	6.3 ± 2 0–9	0.150
	Postop first day	5 ± 3.4 0–10	4 ± 1.6 2–7	0.195
	Postop second day	3.9 ± 3.2 0–10	2.7 ± 1.9 0–8	0.299

Min, minimum; Max, maximum; SD, standard deviation; postop, postoperative.

Group 1 anterior neck VAS (*p* = 0.001, Kruskal–Wallis test) adjusted by Bonferroni correction for multiple tests: a vs. d: *p* = 0.001, a vs. c: *p* = 0.047, b vs. d: *p* = 0.023; group 2 anterior neck VAS (*p* < 0.001, Kruskal–Wallis test) adjusted by Bonferroni correction for multiple tests: e vs. g: *p* = 0.044, e vs. h: *p* < 0.001, f vs. g: *p* = 0.011, f vs. h: *p* < 0.001.

#### Lower chin

**Figure 6:**
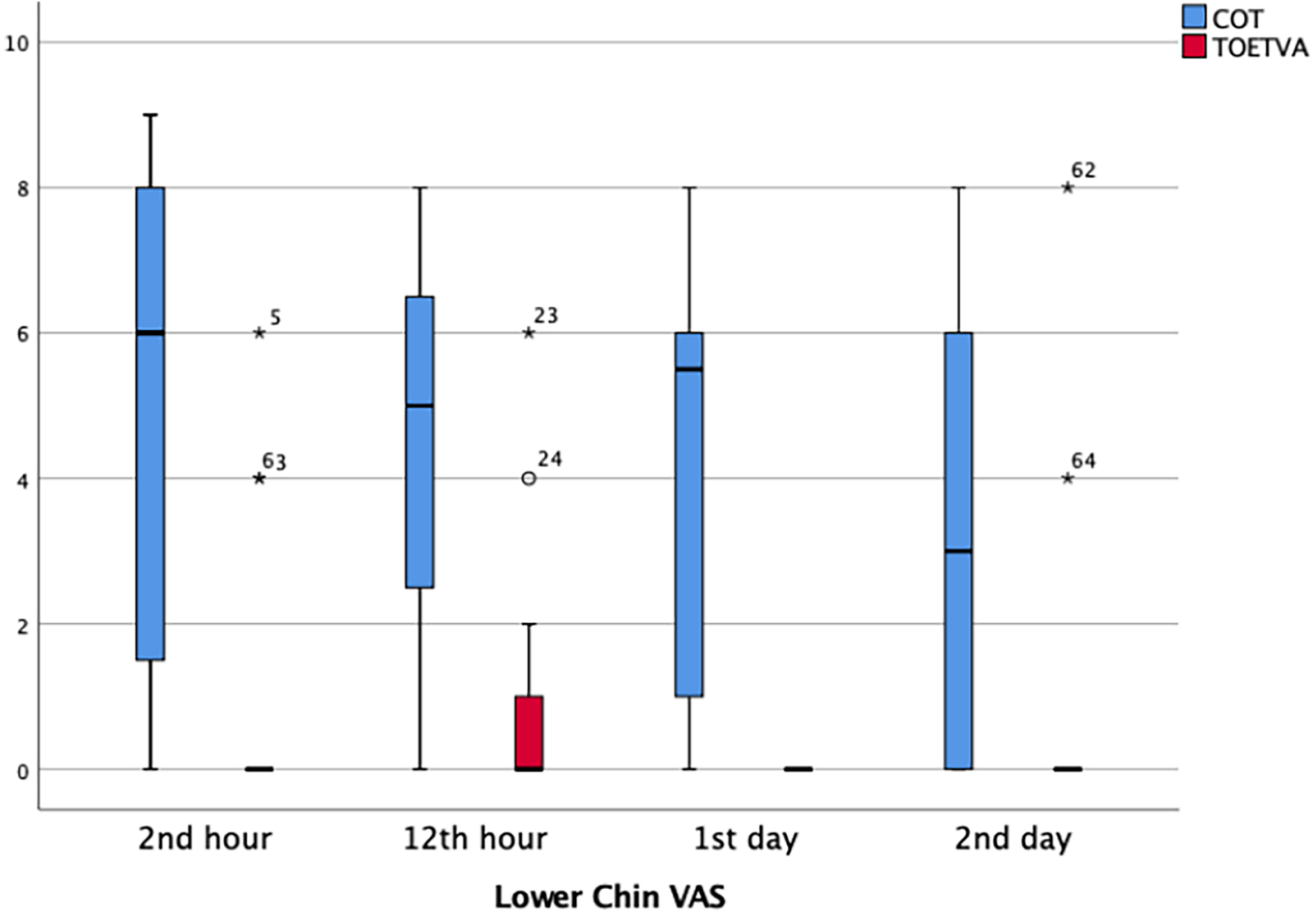
Lower chin VAS score results in graphical illustrations for both groups. *, extreme outlier; o, mild outlier; minimum value that is not an outlier, represented by the bottom of the vertical line (whisker) that extends from the bottom of the box; maximum value that is not an outlier, represented by the top of the vertical line (whisker) that extends from the top of the box; first quartile (Q1), the value below which 25% of the values in the data set are found; median, the value that separates the higher half of the data set from the lower half; third quartile (Q3), the value below which 75% of the values in the data set are found; IQR, the box in the boxplot. This is the middle 50% of the data set (between Q1 and Q3).

Lower chin VAS pain score was significantly higher in the TOETVA compared to the COT group at the second hour (*p* < 0.001), 12th hour (*p* < 0.001), and on the first (*p* < 0.001), and second days (*p* = 0.001) (Table 3) (Figure 6).

#### Anterior neck VAS pain score

**Figure 7:**
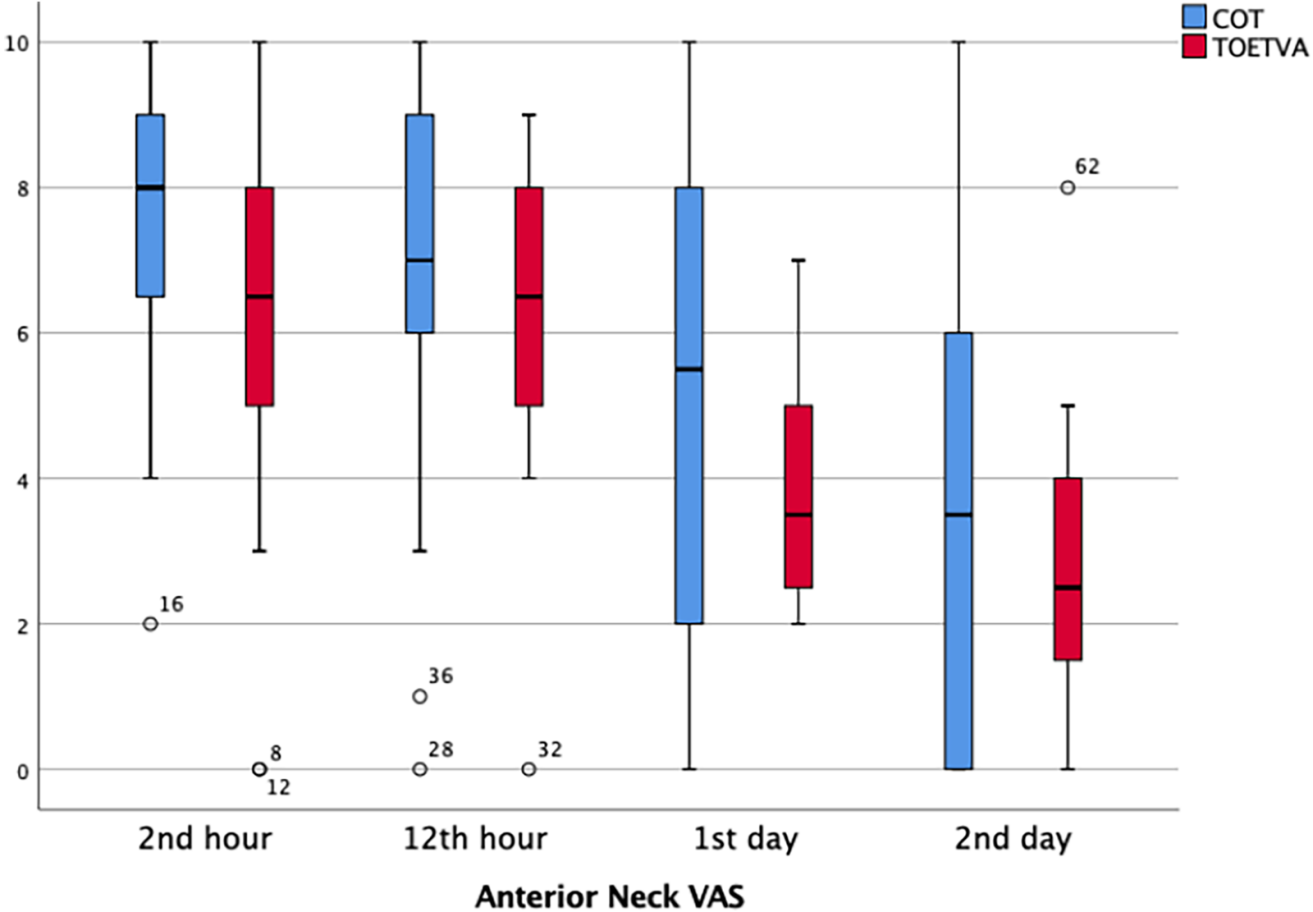
Anterior neck VAS score results in graphical illustrations for both groups. o, mild outlier; minimum value that is not an outlier, represented by the bottom of the vertical line (whisker) that extends from the bottom of the box; maximum value that is not an outlier, represented by the top of the vertical line (whisker) that extends from the top of the box; first quartile (Q1), the value below which 25% of the values in the data set are found; median, the value that separates the higher half of the data set from the lower half; third quartile (Q3), the value below which 75% of the values in the data set are found; IQR, the box in the boxplot. This is the middle 50% of the data set (between Q1 and Q3).

Neck VAS score was significantly higher in the TOETVA group at the postoperative second hour (*p* = 0.025). There was no significant difference regarding the VAS scores between two groups at postoperative 12th hour and on the first and second days (Table 3) (Figure 7). However, it is noteworthy that there was a significant decrease in pain over time in both the COT group and the TOETVA group (*p* = 0.001, *p* < 0.001, respectively).

### Complications

In the TOETVA group, three (13%) patients were excluded because of conversion to open thyroidectomy. In the TOETVA group, three of 20 patients had a skin burn while making the incision for the camera port. In the TOETVA group, loss of sensation on the lower chin or lower lip was detected in 10 patients (50%) in the early postoperative period and decreased over time. Loss of sensation on the chin and lower lip persisted on the 15th day in seven patients and on the 30th day in one patient (Table 4).

**Table 4:** Postoperative complications of the two groups.

	TOETVA	COT	*p*
Hypoparathyroidism (*n*, %)	6 (30%)	5 (25%)	1
Vocal cord paralysis (Number of nerves)			1
Absent	30	31	
Present	3 (9.1%)	3 (8.8%)	
Loss of sensation on the chin			
Postop second day	10	0	<0.001
Postop 15th day	7	0	0.008
Postop first month	1	0	1
Skin burn	3	0	0.231
Wound infection	1	0	1
Tracheal perforation	1	0	1

*n*, number; postop, postoperative.

In the TOETVA group, one patient had tracheal perforation that was recognized preoperatively and repaired by primary suturing. Secondary pneumomediastinum and bilateral pneumothorax were observed postoperatively, and treated with a unilateral thorax tube. Wound infection was detected in the thyroid region of one patient on postoperative sixth day in the TOETVA group, and the collection was percutaneously drained by ultrasound guidance and treated with antibiotherapy. Transient RLN paralysis and hypoparathyroidism rates were similar between the two groups. No permanent RLN paralysis and hypoparathyroidism were detected (Table 4).

There was no significant difference between TOETVA and COT in terms of visual (cosmetic) satisfaction (Table 5). The cosmetic satisfaction score was significantly higher in both TOETVA and COT on the 30th day compared to the 15th day (*p* = 0.003, *p* = 0.003, respectively) (Table 5). There was no significant difference between TOETVA and COT in terms of general satisfaction. General satisfaction scores in both TOETVA and COT were significantly higher on the 30th day compared to the 15th day (*p* = 0.001, *p* = 0.002, respectively) (Table 5).

**Table 5:** Patient satisfaction.

		TOETVA	COT	
Cosmetic satisfaction Mean ± SD Min–max	15th day	3.1 ± 0.8^a^ 2–4	2.9 ± 0.7^c^ 2–4	0.385
	30th day	3.5 ± 0.7^b^ 2–4	3.3 ± 0.5^d^ 3–4	0.160
General satisfaction Mean ± SD Min–max	15th day	3.1 ± 1.1^e^ 1–5	3.7 ± 0.8^g^ 2–5	0.122
	30th day	3.8 ± 1^f^ 2–5	4.2 ± 0.7^h^ 3–5	0.312

Postop, postoperative.

a vs. b: *p* = 0.003, c vs. d: *p* = 0.003, e vs. f: *p* = 0.001, g vs. h: *p* = 0.002.

## Discussion

The application of TOETVA is becoming widespread and accepted as an alternative to traditional thyroidectomy, as a remote access and scarless method in selected cases (18). Although some studies have claimed that TOETVA is a minimally invasive method, discussions are still ongoing whether it is a true minimally invasive method compared to COT or not (9, 11, 19).

Cortisol, IL-6, WBC, and CRP values increase and peak due to SIR after all elective laparoscopic and open surgeries (10). The magnitude of operative injury and invasiveness of surgery after all elective surgeries are associated with only IL-6 and CRP concentrations. Unlike IL-6 and CRP, WBC value is variable, and its relationship with the magnitude of operative injury and invasiveness of the procedure is unclear (10). Therefore, Watt et al. reported that CRP and IL-6 may be used as markers for evaluating and monitoring the magnitude of SIR after elective operations (10).

To the best of our knowledge, the present study is the first prospective one comparing TOETVA with COT in terms of postoperative SIR. WBC, IL-6, and CRP values were monitored to evaluate SIR. In our study, no significant difference was found between preoperative and postoperative IL-6 levels between both groups. Although the postoperative second hour WBC value was significantly higher in the TOETVA group, similar results were obtained on the first and second postoperative days. CRP values were found to be similar preoperatively and at postoperative second hour. At first and second days, although there was a significant increase of CRP in both of the groups, the rate of increase was found to be significantly higher in the TOETVA group. In our study, no drain was used in any patient and no patient required drainage due to seroma. According to our results, the TOETVA operation generates a stronger inflammatory response and is not a minimally invasive method compared to COT. This situation may be related to the fact that more flap dissection is required to reach the surgical site and longer operative time is needed in TOETVA compared to COT.

Sun et al. retrospectively evaluated TOETVA, endoscopic thyroidectomy via areola (ETA), and open conventional thyroidectomy in the treatment of papillary thyroid cancer in terms of SIR. The researchers found that the TOETVA had a higher postoperative CRP and WBC levels than that of the ETA group and the COT group. In addition, the operation time and drainage volume were found to be higher in both TOETVA and ETA groups compared to the COT group. They concluded that although TOETVA and ETA are thought to be more traumatic, these are effective and safe options for the treatment of papillary thyroid cancer (11). In a randomized study comparing the complete endoscopic thyroidectomy via transoral vestibular and areolar approaches, the VAS score on the postoperative first day was lower in the transoral group; however, VAS score on the postoperative third day, CRP, and WBC values were similar (12).

When evaluating the inflammatory response due to operative trauma, potential confounding factors such as age, obesity, comorbid disease, emergency presentation, inflammatory status, and postoperative complications that may affect SIR should also be considered (10). In the last meta-analysis, surgical site infection rate was found higher in TOETVA compared to that in open method (20).

In our study, it can be thought that the postoperative complications such as secondary pneumomediastinum and bilateral pneumothorax caused by intraoperative tracheal perforation in a patient in the TOETVA, and wound infection of another patient may affect the CRP values. When the inflammatory response via CRP is evaluated excluding these two patients, the result did not change, so the data of these patients were not excluded from the study. Although postoperative complications may affect SIR, CRP values on the postoperative second day were similarly increased in the group with and without complications, and CRP values on the postoperative second day were found to be similar (21, 22).

Minimally invasive surgery is generally associated with lower postoperative pain, especially in the abdomen and thorax, compared to open surgery. TOETVA may cause pain complaints in different regions, due to its dissection area, compared to open thyroidectomy. Therefore, regional assessment of pain scores in different regions such as anterior neck, posterior neck, lower lip, chin, and while brushing teeth may be more appropriate (22). We also evaluated the pain symptom separately in the anterior neck region where the incision exists in open thyroidectomy and in the lower chin and lower lip which are the access areas of TOETVA. The same preoperative and postoperative analgesia protocols were applied to both groups of patients. As expected, the VAS score in the lower lip and chin was significantly higher in the TOETVA group compared to open thyroidectomy, and was progressively decreasing in both regions; this is associated with the incision sites and port entry areas. This expected situation is tolerable and has minimal clinical significance. Pain in the neck region was highest at the second postoperative hour and progressively decreased in both groups. Although VAS scores in the TOETVA group was significantly higher only at the postoperative second hour, the difference was not significant in other comparisons. It is noteworthy that neck pain decreased significantly over time in both groups. In another recent study, VAS score was higher in the TOETVA group in the lower lip and chin, and in addition when brushing teeth in the early postoperative period. In the first 24 h postoperatively, contrary to our findings, they found the VAS score lower in the anterior neck and while swallowing in the TOETVA group. It was claimed that less pain was due to more pain receptors in the skin than in the mucosa and less pain receptors in the subplatysmal area in that study. In addition, it has been suggested that due to the different mobility characteristics of the incision sites, patients can better control pain by fixing the inferior vestibular region more easily in TOETVA. They attributed less pain in the posterior neck to less extension in TOETVA than in open surgery (23).

RLN paralysis, hypoparathyroidism, and other rare complications related to thyroidectomy were similar between the two groups. However, TOETVA has revealed new complications such as skin injuries and mental nerve injury. Although Anuwong et al. reported 0.7% transient mental nerve injury, this complication is not uncommon (24). In our study, there was numbness in the access site of the 10 mm port, on the chin or lower lip in the early period, and it decreased over time; it was present in seven patients on the 15th day and in only one patient on the 30th day. This may be related to the temporary loss of function in the sensorial branch of mental nerve branches due to compression at the entry of the midline port and the edema caused by the manipulation of the port during surgery. In a multicentric study involving four centers, numbness was detected in the total lower lip with a rate of 33.6% or of 38.5% in the lower chin, and the incidence ranged between 0% and 100% in different centers. Permanent loss of sensation was detected in only one patient (0.7%), and the mean healing time was 9.2 weeks for the lower lip and 8.1 weeks for the chin tip in that multicentric study (25). Permanent main mental nerve injury can be prevented by applying 5 mm port on both sides of vermillion edge and lateral to the canine tooth and 10 mm port on the middle of the lower lip (9).

Another complication of TOETVA technique is skin puncture and burn marks. In our study, three patients had a burn mark in the mentum developed while entering the camera port. Full-thickness perforations, abrasions, and burns that may develop on the skin can also be the result of hydrodissection with the Veress needle, during port entry or subplatysmal dissection with cautery, or due to energy devices used during thyroidectomy (26).

General and cosmetic patient satisfaction was similar on the 15th and 30th days. In both groups, general and cosmetic satisfaction on the 30th day was significantly higher than that on the 15th day. Surgery-related satisfaction increased over time. The fact that the satisfaction results of the two groups are similar suggests that the performed surgery meets the general and cosmetic expectations of each patient, as they decided on the type of surgery themselves.

The main limitation of the study was the lower mean age in the TOETVA group (42.9 ± 9.7 vs. 50.3 ± 6.2; *p* = 0.008), since it was non-randomized and the surgery type was preferred by the patient. This situation might be considered a limitation in terms of cosmetic evaluation. In addition, the positive and negative aspects of both methods were explained to the patients, and the groups were organized according to the patients’ will in an unbiased manner. Another limitation is that the highest power value has been calculated as 0.623 for the CRP value in the *post-hoc* analysis, which might suggest that the sample size of this study can be considered relatively small. However, prospective studies with larger number of patients are needed.

## Conclusion

In conclusion; the longer operative time, higher postoperative CRP level, and VAS score in the chin and lower lip in the TOETVA group suggest that the method is not a minimally invasive technique compared to COT. However, the presence of similar total complication rates and early postoperative general and esthetic satisfaction, which improves over time in both of the groups, suggest that the clinical effect of increased magnitude of SIR in TOETVA might be temporary and acceptable.

## Data Availability

The original contributions presented in the study are included in the article/Supplementary Material, further inquiries can be directed to the corresponding author.
